# A Cu(II) Indicator Platform Based on Cu(II) Induced Swelling that Changes the Extent of Fluorescein Self-Quenching

**DOI:** 10.3390/polym11121935

**Published:** 2019-11-25

**Authors:** Feifei Wang, Roy P. Planalp, W. Rudolf Seitz

**Affiliations:** Department of Chemistry, University of New Hampshire, Durham, NH 03824, USA; fsq5@wildcats.unh.edu (F.W.); Roy.Planalp@unh.edu (R.P.P.)

**Keywords:** self-quenching, pNIPAM, cross-linked nanoparticles, copper, PA gel

## Abstract

In this study, we established a new fluorescent indicator platform. The responsive element consists of poly(N-isopropylacrylamide) nanospheres that include small percentages of fluorescein and a ligand, anilinodiacetate (phenylIDA). Nanosphere diameters were determined to be in the range from 50 to 90 nm by scanning electron microscopy. They were entrapped in a polyacrylamide gel to prevent nanosphere aggregation. At pH 6, the ligand is negatively charged in the absence of metal ions. Charge-charge repulsion causes the nanosphere to swell. Dynamic light scattering measurements show that these nanospheres do not shrink and aggregate at high temperature. Cu(II) binding neutralizes the charge causing the particles to shrink. This brings fluoresceins closer together, increasing the degree of self-quenching. The intensity decreases by 30% as Cu(II) concentration increases. To rule out the possibility that the observed decrease in intensity was due to Cu(II) quenching of fluorescence, we also added Zn(II) and observed a decrease in intensity. This approach can be adapted to sense different metal ions and different concentrations of Cu(II) by changing the ligand.

## 1. Introduction

Cu(II) is an active producer of oxidative stress for both plants [1,2,3] and animals [4]. Human uptake of Cu is usually in the range of 0.6–1.6 mg per day [5]. Excess uptake of Cu in human beings is related to cancer and aging [5]. It is also reported to be related to diseases of the nervous system such as Alzheimer’s, Menkes, and Wilson diseases [6,7]. Because of its biological effects, control of Cu contamination is an important aspect of environmental protection.

The biotic ligand model (BLM) considers the interactions of all parameters in a natural system to predict the bioavailability of metal ions [8,9]. Bioavailable Cu concentrations predicted by the BLM correlate well with measured Cu LC50s. Total Cu does not correlate well with actual toxicity [9]. However, the BLM is based on an indirect measurement of bioavailable Cu(II), that is, it is based on measurements of organic carbon, pH, other metal ions and several other parameters. At present there is no viable method for measuring bioavailable Cu(II) directly.

Several studies report ligands that change fluorescence when they bind Cu(II). These can potentially be used to measure bioavailable Cu(II). There are some fluorogenic ligands that have increased fluorescence when they bind Cu(II) [10,11,12,13]. However, some of them can only be applied in organic solvents such as THF [10] and acetonitrile [11,14], which are not appropriate for the detection of bioavailable Cu(II) in water systems. Low sensitivity, long response times, poor selectivity and ligands with inappropriate Cu(II)-complex formation constants are other problems that render reported ligands unsuitable for Cu(II) monitoring.

Many other fluorescent sensors have decreased or “turn off” fluorescence upon Cu(II) binding due to Cu quenching of the fluorogenic ligands [15,16,17]. The strategy of developing a fluorogenic ligand that is capable of measuring bioavailable Cu(II) has yet to succeed. Furthermore, even if successful, it would only be applicable to Cu(II).

We prefer to base detection on metal ion induced changes in a water-soluble polymer conformation detected via fluorescence. This approach separates the fluorophore from the metal, rendering it less subject to metal ion quenching, a frequent issue with Cu(II). Furthermore, the selectivity of this approach can be modified by changing the ligand while keeping the rest of the indicator platform. Du et al. synthesized a ratiometric fluorescent Cu(II) indicator platform [18]. Cu(II) binding neutralizes the charge on the ligand, which causes poly(N-isopropylacrylamide) (pNIPAM) to change conformation. This in turn affects the environment of a dansyl comonomer [18]. The indicator developed by Yao et al. [19] is based on fluorescence resonance energy transfer (FRET) [20]. Cu(II) binding introduces positive charge repulsion which separates copolymer strands disrupting FRET. However, neither of these systems has the required sensitivity for environmental Cu(II) measurements. In Du et al.’s indicator, the fluorophore utilized is not that efficient, and for Yao et al.’s indicator, the limit of detection is not low enough. Osambo et al. demonstrated an indicator platform based on changes in FRET accompanying metal ion induced nanoparticle swelling [21]. However, the excitation wavelength is too short to be practical. We also synthesized ratiometric indicators with both donor and acceptor fluorophores on the same polymer chain, but the signal changes with time due to slow polymer untangling. Therefore, our goal is to demonstrate an indicator platform that is both stable and sensitive, and involves wavelengths in the visible spectrum.

**Figure 1:**
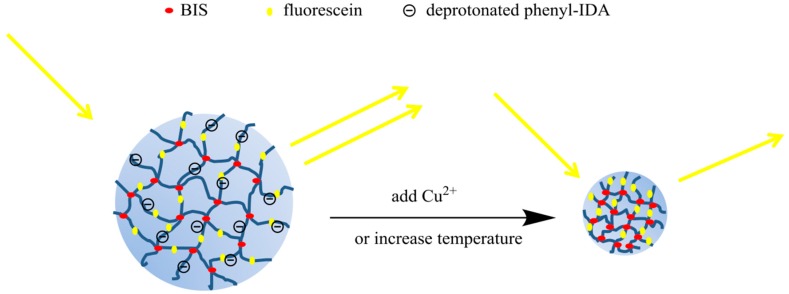
Sensing mechanism of self-quenching poly(N-isopropylacrylamide) (pNIPAM) nanoparticles. Because of the negative charges on the ligand, the nanoparticles swell. When Cu(II) bind to the ligand, the charge neutralization results in less swelling of the nanoparticles, hence the fluorescence intensity also decreases.

The indicator discussed in this paper is based on cross-linked pNIPAM nanoparticles. A negatively charged ligand is used to make the nanoparticle swell in the absence of metal ions. Addition of metal ions neutralizes the negative charge causing the nanoparticle to shrink. This results in a change in fluorescein concentration per unit volume. The fluorescence signal of fluorescein decreases with increasing concentration due to self-quenching when the concentration is above a critical concentration [22]. Our approach is illustrated schematically in Figure 1. However, nanoparticles alone can undergo self-agglomeration, which affects the volume change, and may also block the Cu(II) binding sites. In order to avoid agglomeration, the nanoparticles were embedded in a polyacrylamide gel. The PA gel increases the stability of the single nanoparticles. This approach makes it possible to synthesize particles with a wider range of sizes.

The data we show here are for Cu(II). However, the approach is general because binding of other metal ions will also change the charge on the polymer backbone leading to swelling or shrinking depending on whether the charge increases or decreases. Thus, the indicator platform we demonstrate here is applicable to other metal ions depending on the particular ligand that is incorporated into the polymer.

## 2. Experimental Materials

Materials: Sodium dodecyl sulfate (SDS), *N*-isopropyl acrylamide (NIPAM), *N*,*N*′-Methylenebisacrylamide (BIS), fluorescein *o*-acrylate, potassium persulfate (KPS), acrylamide, ammonium persulfate (APS), *N*,*N*,*N*′,*N*′-Tetramethylethylenediamine (TEMED), Copper (II) nitrate trihydrate, and Zinc (II) nitrate hexahydrate were purchased from Sigma-Aldrich. Aqueous solutions were prepared from doubly distilled water from a Corning Mega-Pure distillation apparatus. Dialysis tubing with a molecular weight cut-off (MWCO) of 3.5–5 kDa was purchased from Spectrum Labs.

Equipment: Fluorescence responses were measured using the scan mode on a Varian Cary Eclipse fluorometer equipped with a Peltier thermostatted single cell holder. Scanning Electron Microscopy (SEM) was performed on a Tescan Lyra3 GMU Focused Ion Beam (FIB) SEM. A Branson model 1800 sonicator was used for reagent dissolution and sonication. A Buchi RE111 Rotavapor was used to evaporate solvents. Separation of precipitated polymer from the solution was performed on a Beckman GP centrifuge (8000 rpm) or an Eppendorf centrifuge 5415 C (14,000 rpm). A FreeZone Plus 2.5 Liter Cascade Benchtop Freeze Dry System was used to lyophilize samples.

## 3. Procedures

### 3.1. The Synthesis of Anilinodiacetic Acid Ligand (Phenyl-IDA) in Ester Form (Tert-Butoxycarbonyl Methyl-(3-Vinyl-Phenyl)-Amino) Acetic Acid Tert-Butyl Ester

**Figure 2:**
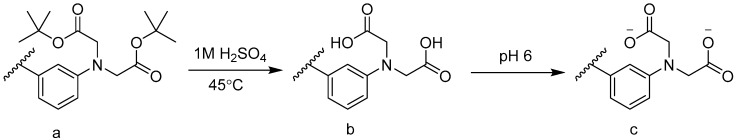
Hydrolysis and deprotonating of phenyl-IDA ligand: (**a**) Phenyl-IDA ester; (**b**) Phenyl-IDA; (**c**) Deprotonating phenyl-IDA.

The esterified ligand was synthesized according to previous literature [21]. The structure is shown in Figure 2. The esterified ligand (compound **a**) was copolymerized into the indicator platform and then hydrolyzed to the acid form (compound **b**). It was deprotonated (compound **c**) in pH 6 buffer.

### 3.2. Emulsion Polymerization of Self-Quenching pNIPAM Nanoparticles

Surfactant, sodium dodecyl sulfate (SDS) (0.14 g, 0.5 mmol) was added to a round bottom flask containing 45 mL deionized water. 1.4 g (12.3 mmol) NIPAM, 0.038 g (0.246 mmol) *N*,*N*′-Methylenebisacrylamide (BIS), 0.005 g fluorescein *o*-acrylate and 0.095 g (0.2 mmol) phenyl-IDA ligand in ester form were added to the mixture under stirring. The solution was stirred and degassed with N_2_ for 30 min. Then the flask was placed in an oil bath at 70 °C. 0.05 g (0.2 mmol) potassium persulfate (KPS) was dissolved in 5 mL DI water, degassed for 5 min and injected into the heated reaction mixture with a syringe. After 6 h, polymerization was quenched by exposure to air. The mixture was dialyzed against deionized water using dialysis tubing with a 3.5–5 kDa molecular weight cut-off with stirring. The water was changed twice daily. After 7 days, the mixture was lyophilized to obtain a pale yellow powder.

### 3.3. Removal of the Ester to Produce the Ligand

The lyophilized nanoparticles with phenyl-IDA were suspended in 50 mL 1 M H_2_SO_4_ solution in a round bottom flask. Acidification (Figure 2) was conducted in an oil bath at 50 °C with stirring for 8 h. Then the mixture was filtered through a glass frit and rinsed with water several times. Another round of acidification was conducted to make sure all of the ligand was hydrolyzed to the acid form. The product was lyophilized and then characterized using a fluorometer and SEM.

### 3.4. Self-Quenching Cross-Linked Nanoparticles Embedded in the PA Gel

Acrylamide (0.475 g), BIS (0.025 g), 250 μL 20× Tris/Borate/EDTA (TBE) buffer and dry pNIPAM particles obtained from the above procedure were added to the 20 mL vial. DI water was added to the vial to bring the volume to 3.83 mL. The solution was degassed for 15 min. Then 20 μL 10% (w/v) APSand 4 μL TEMED were added to the mixture and the solution was gently but thoroughly swirled. The gel solution was quickly and gently introduced into the mold and covered to minimize exposure to oxygen. Gel polymerization proceeded for 2 h. The obtained PA thin film (1 mm) was placed in DI water for 2 days and rinsed with DI water several times in order to remove unreacted monomer and TBE buffer.

### 3.5. Fluorescence Measurement of Nanoparticles Alone

Fluorescence was measured in a 3 mL polystyrene cuvette with 0.1 M pH 6 3-(*N*-morpholino) propanesulfonic acid (MOPS) buffer. This pH keeps the phenyl-IDA ligand in its deprotonated form (compound c in Figure 2) since the pKa_1_ of *N*-Phenyliminodiacetic acid is 2.41 and the pKa_2_ is 5.05 [23]. This pH also prevents Cu(OH)_2_ formation. The particles were suspended in the buffer. The concentration of phenyl-IDA ligand is 10^−5^ M in the cuvette based on the calculations using feed amounts. Cu(II) ions were added from a Cu(NO_3_)_2_ stock solution to the cuvette with a micro pipette in μL, to avoid a significant volume change. Cu(II) concentrations were increased from pCu 7 to pCu 4. Zn(II) ions were added from a Zn(NO_3_)_2_ stock solution to the cuvette with a micro pipette in μL. Since Zn(II) has a lower formation constant with phenyl-IDA, the concentration of Zn(II) was directly brought up to 10^−4^ M to see the response. Both excitation and emission slit widths were 10 nm. The sample was excited at 450 nm. The particles show fluorescein emission at 514 nm.

### 3.6. Fluorescence Measurement of Nanoparticles Embedded in the PA Gel

**Figure 3:**
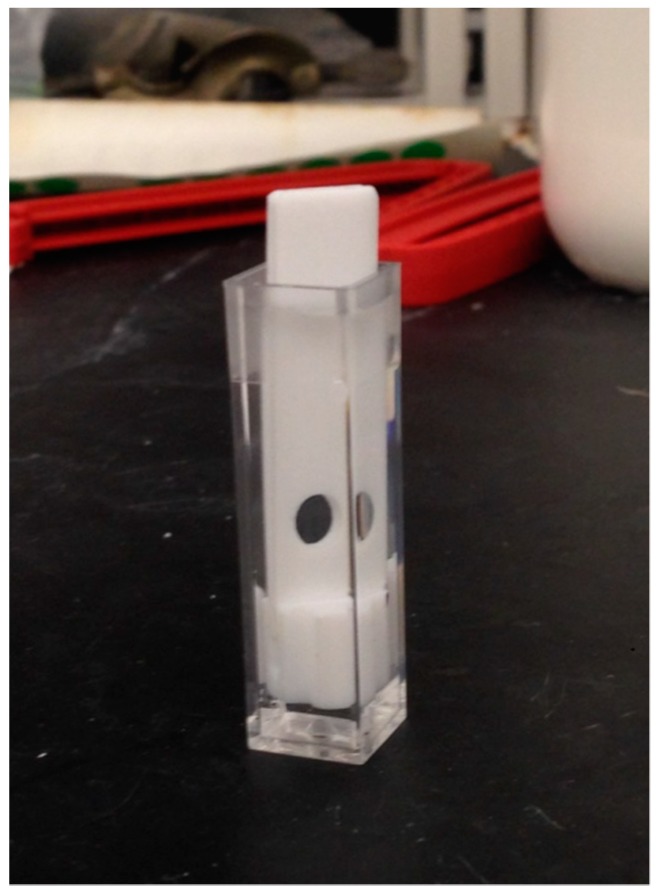
PTFE holder with gel sample at the hole. The height of the hole can be adjusted in order to let incident light go through it and let the detector receive the fluorescence.

The PA gel formed with self-quenching pNIPAM nanoparticles was immersed in DI water and then rinsed several times in order to remove particles that are not immobilized in the gel. The thin film (1 mm) of PA gel sample was fixed using a clean polytetrafluoroethylene (PTFE) holder with a hole so that the gel can completely cover the hole. The location of the hole was adjusted to let incident light go through the gel sample in the fluorometer (Figure 3). MOPS buffer of 0.1 M pH 6 was added to the cuvette. The excitation wavelength was 450 nm. The slit widths were 5 nm. The sample emitted at 514 nm. The theoretical concentration of ligand phenyl-IDA in the cuvette is 10^−5^ M.

## 4. Results and Discussion

### 4.1. Morphology of the Nanoparticles

Scanning electron microscopy (SEM) was used to study the morphological features of self-quenching pNIPAM particles. The sample was taken from the stock suspension of 0.1 g/L prepared with the lyophilized powder. A platinum sputtering layer was coated onto the sample stub after the sample was dried.

**Figure 4:**
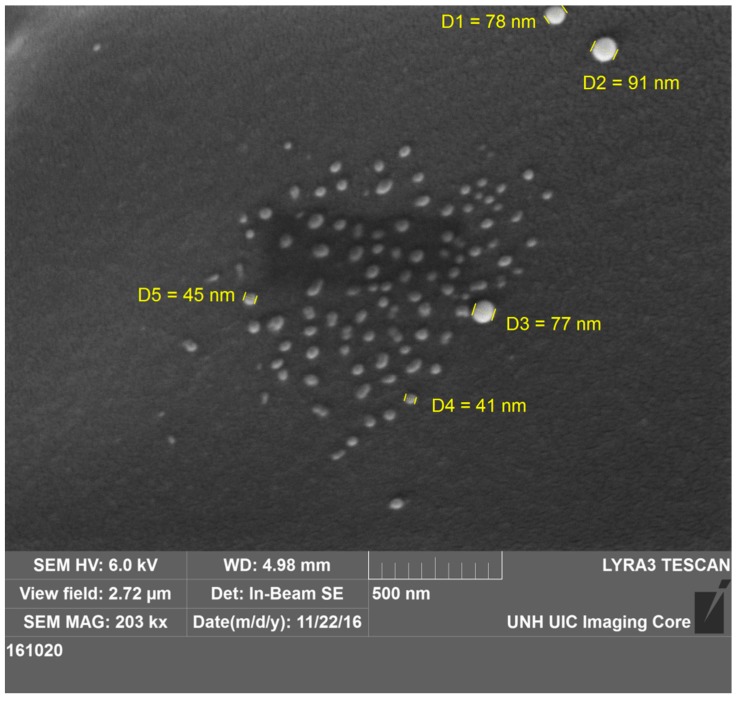
SEM image of self-quenching pNIPAM nanoparticles. The sizes of the particles range from 40 to 90 nm.

The size of the dry particles ranges from 40 to 90 nm (Figure 4). The surface of the particles is not that smooth, and there is some deformation in shape. Some of this is the result of two particles merging to form a larger particle. There is not much agglomeration of nanoparticles, probably because of the dilute solution.

### 4.2. Fluoresence Study of the Self-Quenching pNIPAM Nanoparticles Alone

#### 4.2.1. Thermal Response

**Figure 5:**
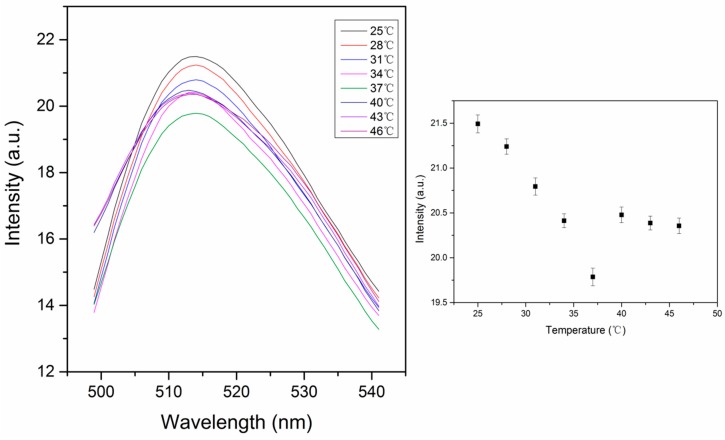
Thermal response of self-quenching pNIPAM nanoparticles from 25 °C to 46 °C. The intensity values in the right figure were taken from the peak intensity in the left figure. (ex: 450 nm, slit widths: 10 nm).

PNIPAM has reverse solubility upon heating. The thermal response is characterized by a lower critical solution temperature (LCST), where the pNIPAM abruptly transitions from hydrophilic to hydrophobic. This occurs because hydrogen bonding gets weaker with increasing temperature, reaching a point where it is no longer able to prevent hydrophobic collapse [24]. The LCST of pNIPAM is in the range of 30–35 °C [25]. When the polymer chains are cross-linked, the polymer is swollen with water below the LCST and collapses, excluding water above the LCST. Our pNIPAM nanoparticles are prepared with 2 mol% BIS, 2 mol% phenyl-IDA, 0.2% (w/v) fluorescein *o*-acrylate and NIPAM, by emulsion polymerization in order to control the diameter of the particles. The concentration of fluorescein in the particles was set at 0.2 g per 100 g of nanoparticles, which is the critical concentration for self-quenching [22]. This is based on the assumptions that the density of the nanoparticles is 1 g/mL. Therefore we can get high fluorescence intensity and significant self-quenching simultaneously. The fluorescence signal decreases with increasing temperature from 25 °C to 46 °C (Figure 5). This thermal response could be from both thermal quenching and particle shrinking that leads to more self-quenching. When the temperature increases, pNIPAM shrinks, causing the fluorescein to be closer to each other, which leads to self-quenching. Our data show only a small decrease in fluorescence with temperature with no sign of a large change that would be indicative of the thermal phase transition. From this we conclude that the presence of phenyl IDA in the polymer chain is preventing hydrophobic collapse of the cross-linked nanoparticles. This is consistent with our expectation that we have removed the t-butyl groups leaving carboxylates that are deprotonated at the pH of this measurement. The literature pKa_1_ of *N*-Phenyliminodiacetic acid is 2.41 while pKa_2_ is 5.05 [23], so in the pH 6 MOPS buffer the phenyl IDA ligand is deprotonated to produce negative charges. Based on the acid-base equilibrium, the fraction of the deprotonated form of ligand can be estimated using Equation (1):

A = [L^2−^]/c_L_ = Ka_1_Ka_2_/([H^+^]^2^ + Ka_1_[H^+^] + Ka_1_Ka_2_) (1)

where α is the fraction of deprotonated form of phenyl IDA ligand that has two charges, c_L_ is the total concentration of ligand. The fraction α of phenyl IDA with two charges is 89.9% in pH 6 buffers. That means the rest of the ligand should have one charge. This calculation assumes that the published solution pKa values for phenyl IDA apply to phenyl IDA that has been copolymerized with NIPAM. In practice, this is unlikely. However, given the small value of pKa_1_, we feel safe in assuming that essentially all the immobilized ligand is charged.

#### 4.2.2. Response to Cu(II)

**Figure 6:**
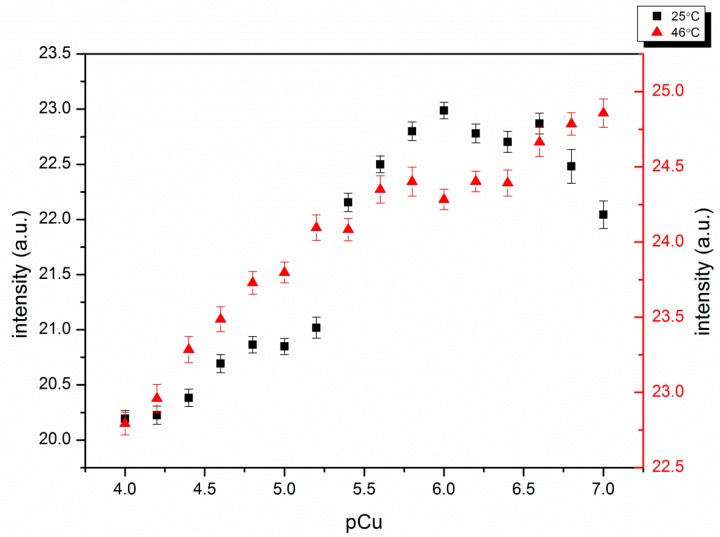
Cu(II) response of self-quenching pNIPAM nanoparticles alone at 25 °C and 46 °C. pCu = −log [Cu^2+^]. Smaller pCu represents higher Cu(II) concentration.

Cu(II) quenches fluorescence when bound to a fluorophore because it is paramagnetic [26]. This system avoids the problem by exploiting a polymer conformational change that leads to a change in the emission from the fluorophore. The synthesized self-quenching pNIPAM nanoparticles exhibit decreased fluorescence with increasing Cu(II) concentration, below and above the LCST (25 °C and 46 °C) (Figure 6). This occurs when Cu(II) binding neutralizes the negative charges, causing particles to shrink and the self-quenching of fluorescein to increase. There are big changes around pCu = 5.3. This occurs when Cu(II) concentration is close to the ligand concentration, which is estimated to be 10^−5^ M in the buffer based on initial amounts in the polymerization. This is sufficient to neutralize all the charges on the polymer. The log Kf for Cu(II)-phenyl-IDA is 6.37, large enough so that essentially all the Cu(II) is bound to ligand when the concentrations of both are close to 10^−5^ M. When the ligand is on the polymer, water has less access to the charges on the ligand to stabilize them. Because of this we expect the log Kf for Cu(II) phenyl-IDA to be somewhat larger than 6.37 for the polymer bound ligand.

The concentration of the nanoparticles in the cuvette was 0.01 g/L in order to get an easily measurable signal while still avoiding particle aggregation. The measurements of the response to Cu(II) were taken within 10 min, and at either temperature, there was no visible aggregation. This relative stability can be ascribed to the negative charges on the ligands. It is expected that the uncharged nanoparticles may aggregate when the temperature is above the LCST.

### 4.3. Fluorescence Study of the Self-Quenching pNIPAM Nanoparticles Embedded in the PA Gel

The intensity change shown in Figure 6 is not as large as we expected. The possible cause is that there is minor aggregation, which inhibits the volume change upon Cu(II) binding. In order to prevent aggregation, an embedding medium was developed. Polyacrylamide (PA) is a cross-linked gel that is commonly used for polyacrylamide gel electrophoresis. PA gel is transparent, relatively chemically inert and the pore size can be controlled [27]. These features make it not only a good support in electrophoresis, but also a potential embedding medium for biological functional units like enzymes, antibodies, and synthetic agents or particles [28,29]. The self-quenching pNIPAM nanoparticles were embedded in a PA gel in order to prevent possible particle aggregation and increase the signal change.

The pore size of the gel was controlled to 3.4–34 nm by choosing the appropriate total monomer concentration and weight percentage of cross-linker [30]. The pNIPAM nanoparticles were mixed with the gel solution before polymerization and were trapped in the gel after gel formation.

**Figure 7:**
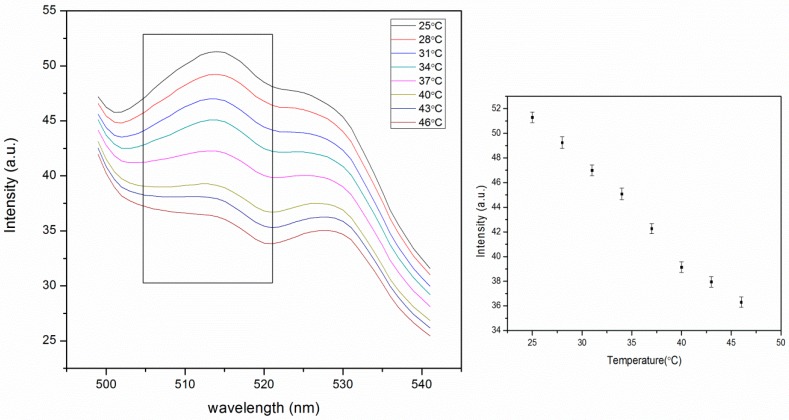
Thermal response of self-quenching pNIPAM nanoparticles embedded in the polyacrylamide (PA) gel from 25 °C to 46 °C. The intensity values in the figure on the right were taken from the peak intensity at 515 nm in the figure on the left. The tailing before 500 nm is from the background scattering of the PA gel. The peak at 530 nm is from the fluorescence of PTFE holder. (ex: 450 nm, slit widths: 5 nm).

The fluorescence intensity of the embedded nanoparticles continuously decreases with increasing temperature (Figure 7). The higher signal is due to less aggregation. This change is much larger than that of pNIPAM particles alone (Figure 5). This is due to the higher stability of particles in the PA gel where they cannot aggregate, which affects the signal. The thermal phase transition of pNIPAM can be observed as the slight slope change around 37 °C in the graph of fluorescence intensity vs. temperature (Figure 7). This is consistent with the response of pNIPAM particles alone (Figure 5). The decrease in the fluorescence intensity is very large, from 51 to 36 a.u as the temperature increases from 25 to 46 °C. In a separate experiment we determined that the fluorescence of fluorescein decreases by approximately 1.1% per degree C for fluorescein in pH 6 buffer. This means that the decrease observed in Figure 7 is greater than the temperature effect on fluorescein and, therefore, presumably involves a degree of increased selfquenching due to particle shrinking. The change is 30%, much larger than 5%, the change for particles only, as shown in Figure 5.

**Figure 8:**
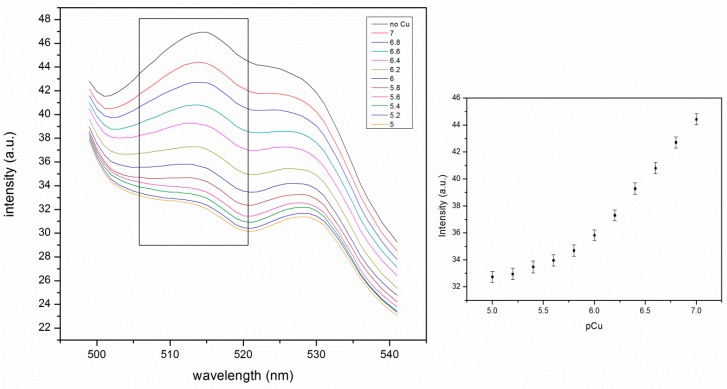
Cu(II) response of self-quenching pNIPAM nanoparticles in the PA gel at 25 °C. The intensity values in the figure on the right is taken from the peak intensity at 515 nm in the figure on the left (ex: 450 nm, slit widths: 5 nm).

The Cu(II) induced response of the embedded pNIPAM nanoparticles at 25 °C is also much larger than that of the pNIPAM particles alone. The fluorescence intensity drops from 44 to 33 a.u. This is due to the neutralization of the negative charges of phenyl-IDA by Cu(II) binding (Figure 8). Charge neutralization allows the particles to shrink, thus causing more fluorescein self-quenching.

In a control experiment, we determined that Cu(II) concentrations as high as 0.001 M did not quench fluorescein fluorescence when Cu(II) was added to solutions of fluorescein in pH 6 buffer. This rules out the possibility of quenching by solution phase Cu(II) as an explanation for the observed intensity decrease.

The shapes of the fluorescence spectra of PA gel supported nanoparticles (Figure 7 and Figure 8) are different from those of nanoparticles alone (Figure 5). This is due to some background scattering from the gel. The excitation wavelength was fixed at 450 nm in order to avoid the overlapping of the scattering peak from water with the fluorescein peak.

The metal ion response is slow because diffusion of metal ions into a PA gel takes more time than binding to the pNIPAM particle alone. Because the pore size (3.4~34 nm) of the PA gel is much larger than Cu (II), and PA has excellent hydrophilicity, the absorption of Cu(II) should be rapid. If the pINPAM particles in the gel are evenly distributed, the metal ions are absorbed into the gel and bind to the ligand. The binding of Cu(II) to the ligand may also be slowed down by the interaction with the gel. The time it takes to reach equilibrium is hard to estimate. Therefore the data were collected when the signal stabilized. The time it took to stabilize was about 10 min for each set of data.

The high signal intensity in Figure 8 is due to the stability provided by the PA gel. The nanoparticles stay apart, leading to less self-quenching from adjacent particles. The concentration of the particles in the gel can be decreased to a much lower level than 0.01 g/L with increased slit width. This is important for the application to environmental monitoring since the concentration of the indicator needs be lowered so that the presence of ligand does not perturb the natural system.

### 4.4. Zn(II) Responses of Self-Quenching pNIPAM Nanoparticles Alone and Particles Embedded in the PA Gel

**Figure 9:**
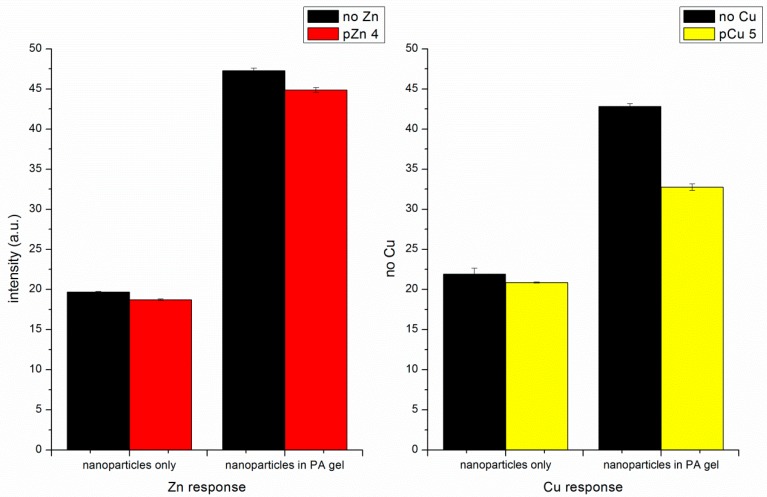
Comparison of Zn(II) response and Cu(II) response of self-quenching pNIPAM nanoparticles alone and particles embedded in the PA gel.

To confirm that the decrease in fluorescence intensity is mainly the result of the volume change rather than Cu(II) quenching, fluorescence measurements with Zn(II) addition were conducted. Unlike Cu(II), Zn(II) does not quench fluorescence. Zn(II) ions were added from a Zn(NO_3_)_2_ stock solution to the cuvette. Both the pNIPAM nanoparticles alone and the embedded pNIPAM nanoparticles show decreased fluorescence signal upon Zn(II) addition (Figure 9). This response confirms that the metal ions added to the particle cause a volume change, while some of the observed response to Cu(II) may be due to quenching. We also see a decrease in intensity due to increased fluorescein self-quenching.

The literature formation constant for Cu(II)-phenyl IDA is log K_f_ = 6.37 while that for Zn(II)-phenyl IDA is log K_f_ = 3.53 [31]. The ligand phenyl-IDA has much lower affinity towards Zn(II). With the same level of indicator present, the Zn(II) addition may not have the same effect as Cu(II) addition because the Zn(II) does not completely bind to the ligand. The decrease in intensity with Cu(II) (Figure 6 and Figure 7) is larger than with Zn(II) (Figure 9).

## 5. Conclusions

A fluorescent metal ion indicator based on cross-linked pNIPAM nanoparticles was synthesized by copolymerizing fluorescein, ligand phenyl-IDA and NIPAM and cross-linker. The negative charges on the ligand make the nanoparticle swell. When Cu(II) ions are added to the system, they bind to the ligand and neutralize the negative charges, decreasing the swelling extent. The shrinkage of the particles leads to a shorter distance between adjacent fluoresceins, thus increasing self-quenching. The fluorescence intensity decreases with increasing Cu(II) concentration. Embedding the nanoparticles in the PA gel causes a larger change in the quenching due to shrinkage.

This indicator platform has several advantages over other platforms: (a) This indicator responds to Cu(II), which normally quenches fluorescence. The separation of ligand and fluorophores makes the binding site of Cu(II) separate from the fluorophore, thus decreasing the paramagnetic quenching effect on fluorescence. (b) The sensitivity and selectivity of the indicator can be modified by utilizing different ligands without changing the excitation wavelength. Theoretically, the indicator platform responds to all metal ions with appropriate ligands. (c) It was reported that a cross-linked structure improves the thermal stability of polymers [32], and in this indicator platform, it also helps to solve the problem of the stability of free-floating or end-grafted polymer chains, since the untangling of the polymer chains is not an issue. The nanoparticles are also easy to purify and recycle by centrifugation. (d) The self-quenching pNIPAM nanoparticles were embedded in a PA gel in order to prevent possible particle aggregation and increase the signal change.

The ultimate purpose of the indicator is to measure bioavailable metal ions in the environment. Future work may involve the application of a reference fluorophore in the gel to obtain ratiometric measurements, which can reduce error due to instrumental drift and simplify calibration. Meanwhile, we also plan to try an array with a donor fluorophore on one end of the pNIPAM chain and an acceptor fluorophore on the other end, to get a much larger signal change. The sensitivity can also be improved by using fluorophores with high quantum yield, and the limit of detection can be improved with high Cu(II) affinity ligands, which enables accurate readouts with low indicator concentration. This is beneficial to environmental monitoring since the equilibria in the environment would not be disturbed by the indicator. Moreover, the indicator is expected to selectively respond to target metal ions without the interference of other metals when metal-selective ligand systems are incorporated into the polymer.
